# Outcomes of iontophoretic corneal collagen crosslinking in keratoconic eyes with very thin corneas

**DOI:** 10.1097/MD.0000000000008758

**Published:** 2017-11-27

**Authors:** Alina Cantemir, Anisia-Iuliana Alexa, Bogdan Gabriel Galan, Nicoleta Anton, Roxana Elena Ciuntu, Ciprian Danielescu, Dorin Chiselita, Danut Costin

**Affiliations:** a Grigore T. Popa University of Medicine and Pharmacy; b Sanoptic Eye Center, Iasi, Romania.

**Keywords:** iontophoretic corneal collagen crosslinking, keratoconus, pachymetry, riboflavin, thin cornea

## Abstract

The purpose of this retrospective study was to report the results of iontophoretic corneal collagen crosslinking (I-CXL) with riboflavin and ultraviolet A irradiation in patients affected by keratoconus, each with thinnest pachymetry values of <400 μ (with epithelium) and not treatable using standard epithelium-off technique.

Fifteen eyes of 15 patients affected by progressive keratoconus and with thinnest pachymetry values <400 μ underwent I-CXL. The uncorrected (UDVA) and corrected (CDVA) distance visual acuity, maximum and minimum keratometry (K max and K min) readings, corneal thickness at the thinnest point (CTTP), endothelial cell density (ECD), and intraocular pressure (IOP) were assessed before I-CXL, at 1, 3, 6, and 12 months postoperatively.

The mean UDVA and CDVA significantly increased 12 months after I-CXL (*P* = .002 for both comparisons). The K max readings significantly decreased at 6 and 12 months postoperatively (*P* = .04 and *P* = .02, respectively). The mean CTTP improved at the end of the follow-up (*P* = .008). ECD was unchanged. No side effects or damage to the limbal region was observed during the follow-up period.

I-CXL has been proved to be effective in halting keratoconus progression in eyes with very thin corneas, with no side effects during the follow-up period. This procedure could be used in patients with more advanced keratoconus.

## 1 Introduction

Keratoconus is the most common corneal dystrophy, with a frequency of about 1 to 2000, manifested by progressive thinning and deformation of the cornea, resulting in myopia and irregular astigmatism.^[1]^

Corneal collagen crosslinking (CXL) is a proved efficient procedure in halting the progression of keratoconus in both laboratory and clinical trials with up to 10 years of follow-up.^[2,3]^ The standard crosslinking technique involves removing the epithelium to allow the riboflavin to penetrate into the stroma, and subsequently, under the action of ultraviolet radiation A, a photopolymerization reaction of the collagen fibers from the corneal structure to occur.^[4,5]^ An increase in the number of covalent intra- and interfibrillary bonds that form a dense network has been shown, thus increasing the corneal rigidity as well as halting the disease progression.^[6]^

The standard CXL has been proved to be a safe method on short and long term with no side effects due to irradiation on the corneal endothelium, the lens or the retina, as long as the corneal stromal thickness is >400 μ.^[2,3,7]^ Since patients are often diagnosed at advanced stages of disease with pachymetry below 400 μ and the standard CXL is contraindicated, different alternative crosslinking techniques have been proposed: transepithelial CXL with an improved riboflavin with 15% dextran, EDTA, and trometamol,^[8]^ the use of a hypoosmolar solution of riboflavin,^[9]^ customized epithelial debridement according to pachymetry,^[10]^ and disruption of the corneal epithelium with a special instrument in combination with administration of hypotonic riboflavin.^[11]^

The iontophoretic corneal collagen crosslinking (I-CXL) is a new, promising technique that uses low-intensity electric current to facilitate the penetration of riboflavin, a low molecular weight substance electronegatively charged, through the intact corneal epithelium.^[12,13]^

Preclinical studies have shown that I-CXL leads to a deeper penetration of riboflavin into the corneal stroma than the transepithelial CXL, producing histological changes and resulting in corneal stiffening comparable with standard CXL.^[14]^ A relatively small number of clinical studies have demonstrated the efficacy and safety of iontophoretic crosslinking in both adults and the pediatric population, in halting the disease progression in most cases, with a rapid vision recovery and virtually with no side effects.^[15–19]^

## 2 Aim

In this study, we aimed to evaluate the effectiveness and safety of I-CXL in patients with keratoconus with corneal thickness values at the thinnest point (CTTP) <400 μ (with epithelium). We reported the evolution of visual results, refractive and topographic parameters, of intraocular pressure (IOP) as well as the endothelial cell density (ECD) 12 months after I-CXL.

## 3 Methods

### 3.1 Patients

In this clinical, retrospective study we included 15 eyes of 15 patients with progressive keratoconus, with very thin cornea (CTTP <400 μ, with epithelium) who were treated at the Oftaprof Clinic of Iasi, Romania, between January 1, 2012 and January 1, 2015. The patients were identified using the computerized clinic record system; the follow-up period was 1 year for each patient. Keratoconus diagnosis and the necessity of treatment were assessed by 2 corneal specialists (AC, DC).

The study was approved by Oftaprof Clinic ethics committee and it was conducted accordingly to the ethical standards set in by the 1964 Declaration of Helsinki, as revised in 2003. Written informed consent was obtained from all patients at the time of the procedure.

The inclusion criteria were age above 18 years, progressive keratoconus with CTTP pachymetry <400 μ, and the ECD >2000 cell/mm^2^.

Patients who had been diagnosed with diabetes, immune system diseases, and other ocular and corneal disorders (herpetic keratitis, dry eye syndrome, and corneal scarring), or CCTP >400 μm were excluded from the study. Other exclusion criteria were pregnancy or breastfeeding.

### 3.2 Visits

All patients were examined at baseline and 1, 3, 6, and 12 months after the intervention. The following measurements were performed at baseline and at each follow-up visit: slit-lamp examination of the anterior segment of the eye, uncorrected distance visual acuity (UDVA, logMAR units), corrected distance visual acuity (CDVA, logMAR units), manifest refraction (expressed as sphere and cylinder, diopters, D), maximum and minimum keratometry (K max and K min, diopters, D) assessed by optical tomography (Pentacam, Oculus Optikgeräte GmbH, Wetzlar, Germany), ultrasonic pachymetry for corneal thickness at the thinnest point, ECD (Konan Specular Microscope; Konan Medical, Inc, Hyogo, Japan), and IOP by Goldmann applanation tonometry. To improve the quality of topographic parameters, at least 3 acquisitions per eye resulted in all examinations. The best scan was taken into account for analysis. The postoperative demarcation line depth was measured 1 month after intervention by anterior segment optical coherence tomography (AS-OCT, Visante OCT; Carl Zeiss Meditec, Jena, Germany).

### 3.3 Surgical technique

Collagen cross-linking by iontophoresis was performed for 15 eyes (15 patients) included in the study. Pilocarpine 1% drops were instilled 30 minutes before the procedure, to reduce the risk for ultraviolet light exposure of the lens and retina. The procedure was performed under topical anesthesia with 3 applications of 4% lidocaine drops under sterile conditions during 15 minutes, using a commercial iontophoresis device (Iontophor CXL, Sooft Italia SpA, Italy). A special ring applicator containing the active electrode (stainless steel mesh) was applied to the corneal surface, and a patch containing the passive electrode was applied to the forehead. A continuous current generator (I-ON XL, SOOFT) set at 1 mA, powered by batteries, was connected to the relevant cables. The applicator was filled with 0.5 mL hypotonic solution of Riboflavin (Ricrolin+; SOOFT, Montegiorgio, Italy) and the corneas underwent imbibition for 5 minutes. After iontophoresis, the corneal surface was washed with 0.9% sodium chloride solution. The corneas were irradiated with an ultraviolet lamp (VEGA CBM-X-Linker, CSO, Firenze, Italy) with a wavelength of 370 nm for 9 minutes at 10 mW at a distance of 45 mm. After surgery, a bandage contact lens was applied and it was removed after 48 hours. Post-intervention, patients received tobramycin-dexamethasone drops (Tobradex, Alcon) and artificial tears (Blu Yal, SOOFT), 4 times daily, for 4 weeks.

### 3.4 Statistical analysis

The statistical analysis was performed using the SPSS statistical software for 20.0 Windows version (SPSS, Inc, Chicago, IL). Data are described as mean ± standard deviation. A *P* value of <.05 was considered significant in all cases. Decimal CDVA was converted to logMAR units (the logarithm of the minimum angle of resolution) for statistical analysis.

The data in our cohorts do not have a normal distribution (according to Kolmogorov-Smirnov and Shapiro-Wilk tests). The Wilcoxon test was used to evaluate the significance of differences between preoperative and postoperative data.

## 4 Results

The study included 15 eyes of 15 patients, with an average age of 26.4 ± 3.23 (18–34) years. Most patients were male (9 patients, 60%). Epithelial healing was complete in all patients after 1 day of use of a soft bandage contact lens.

UDVA remained unchanged during the first 3 months postoperatively, with a significantly statistical increase both at 6 and 12 months (*P* = .007 and *P* = .002, respectively). At 12 months postoperatively, the mean UDVA increased by 0.7 Snellen lines (Table 1).

**Table 1:**
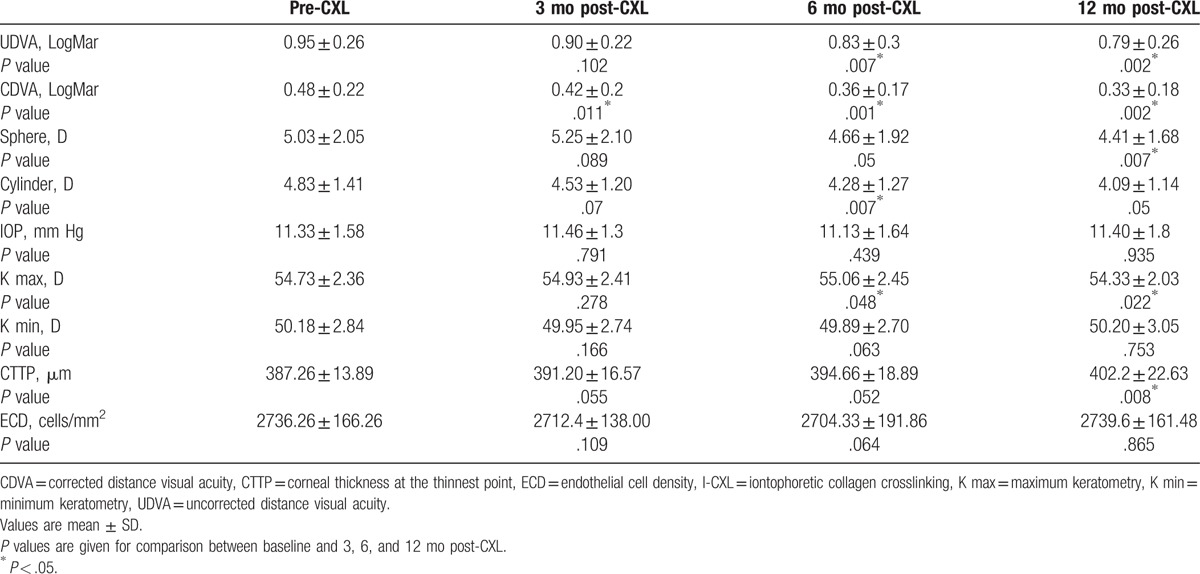
Clinical outcomes of I-CXL for very thin corneas.

**Figure 1:**
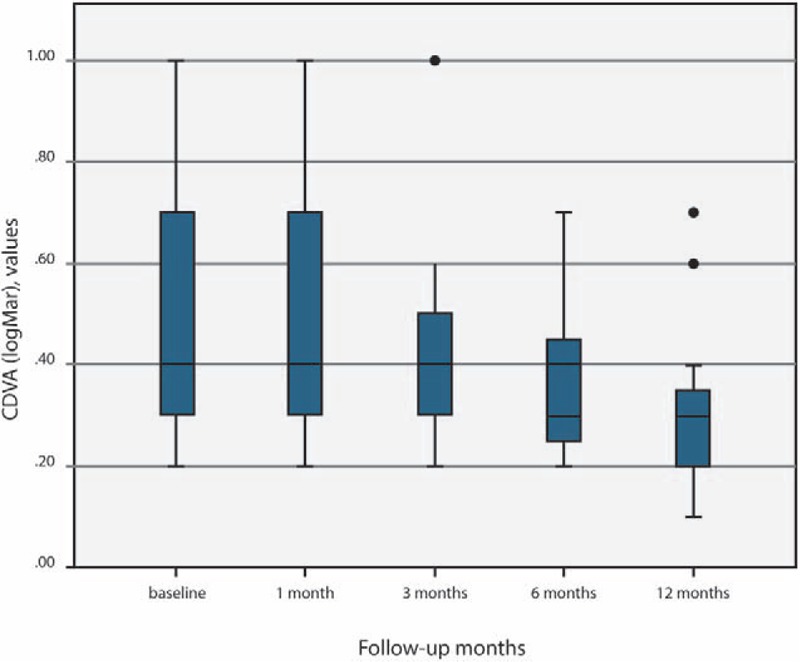
CDVA readings comparing with baseline at 1, 3, 6, and 12 mo after I-CXL.

CDVA remained unchanged in the first month postoperatively, with a significantly statistical increase during the 6 and 12 months postoperative evaluations (*P* = .001 and *P* = .002 respectively). At 12 months postoperatively the mean CDVA increased by 0.9 Snellen lines (Table 1 and Fig. 1).

**Figure 2:**
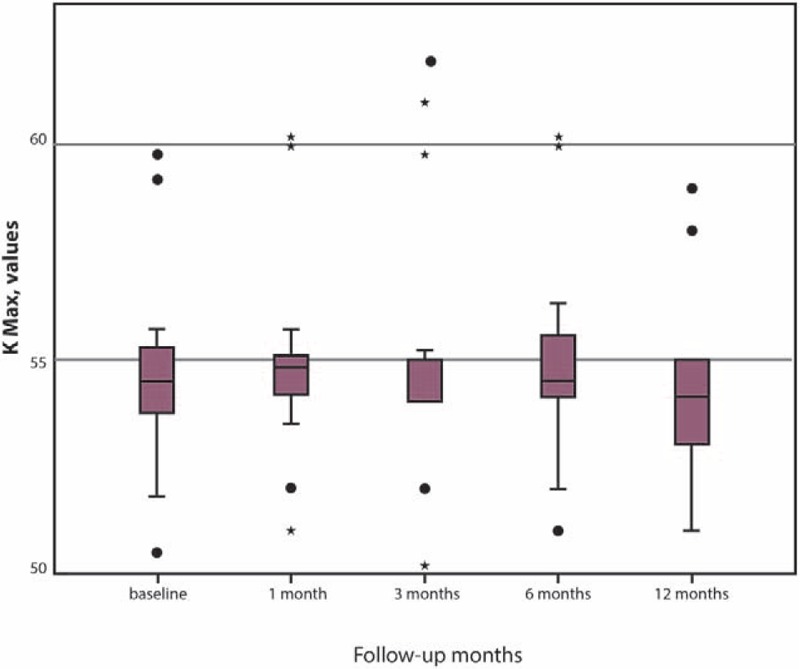
K max readings comparing with baseline at 1, 3, 6, and 12 mo after I-CXL.

The mean K max remained stable in the first 3 months postoperatively, a statistically significant decrease being evident during the 6 and 12 months postoperative evaluations (*P* = .04 and *P* = .02, respectively) (Table 1 and Fig. 2).

The mean value of K min was unchanged from baseline over the entire follow-up period (Table 1).

The refraction was unchanged in the first 6 months followed by a statistically significant decrease of the mean value of the sphere (from 5.03 D to 4.4 D, *P* = .007 at 7 to 12 months) and that of the cylinder (from 4.83 to 4.09 D, *P* = .005 at 12 months) (Table 1).

There was a slight increase in CTTP beginning with the third postoperative month; this reached the threshold of statistical significance in the 12th postoperative month (*P* = .008) (Table 1).

A clear demarcation line was identified in only 5 cases (30%) of cases with an average depth of 184 ± 26 μ; most patients experienced only an increase in the reflectivity of the anterior stroma. There were no statistically significant changes in IOP and ECD throughout the study (Table 1).

No side effects or damage to the limbal region was observed during the follow-up period.

## 5 Discussion

In this study we examined 1-year outcomes of iontophoretic corneal crosslinking in the treatment of progressive keratoconus with very thin cornea, below 400 μ. Our results are encouraging to stabilizing the disease, improving functional and refractive parameters, and the safety of the procedure.

CXL is the standard method of treatment that inhibits the progression of keratoconus.^[5,6]^ The procedure is used in cases where the thickness of the cornea is over 400 μ, where a substantial part of the stroma thickness is treated to obtain the corneal stiffening effect without endangering the corneal endothelium, the lens and other ocular structures.^[4,20]^ The standard CXL technique is not safe when the corneal thickness is <400 μ; in these cases, new techniques have been tried to safely halt the disease progression.

There are few studies that analyze the crosslinking results in patients with a corneal thickness of <400 μ.

Cagil et al mark the corneal areas thinner than 400 μ (based on topographic and pachymetric measurements) and remove the corneal epithelium outside these areas on 8.5 mm diameter area. The authors use this procedure in 19 patients followed for 12 months and find the following: UDVA amelioration and a significant decrease in K max and K min but with the reduction in the mean of ECD, without altering pleomorphism and cellular polymegethism. The authors conclude that this method is capable of halting the progression of keratoconus with thin corneas.^[21]^

Spadea and Mencucci use the transepithelial CXL technique where the corneal epithelium penetration is improved by the use of riboflavin 0.1% solution in 15% dextran containing 0.01% EDTA and trometamol. Sixteen patients with ultrathin cornea were evaluated and the postoperative follow-up was 12 months. The authors observed a slight improvement in UDVA and CDVA, a small decrease in keratometric astigmatism, without evident changes in ECD. The authors admit that this procedure is safe and has moderate effectiveness in the eyes with keratoconus and ultrathin cornea.^[22]^

Hirji et al use a technique that associates corneal epithelial disruption (using a specific surgical instrument) with corneal stroma saturation by a 0.1% riboflavin hypotonic solution without dextran. In the group of patients with thin cornea (15 eyes) followed for 12 months, they found a K max reduction of approximately 1.5 D, an improvement of CDVA with at least 2 Snellen lines in 25% of cases, and stabilization in 75% of cases without any change in the corneal thickness and ECD. They demonstrate that the corneal epithelial disruption is safe and allows good diffusion of riboflavin in the corneal stroma.^[11]^

I-CXL allows the preservation of corneal epithelium, increases riboflavin concentration within the corneal stroma more than in transepithelial CXL and reduces the treatment time.^[13,23,24]^

The rationale for using I-CXL in patients with progressive keratoconus and very thin cornea is related to several factors. First, its safety and efficiency has been proved when the corneal thickness is over 400 μ. The preservation of the corneal epithelium increases patients’ comfort, accelerates visual rehabilitation, and reduces the risk of complications (infectious keratitis, stromal infiltrates, and corneal thinning and melting).^[25,26]^ Second, it is about the depth of riboflavin penetration into the corneal stroma. It is admitted that the stromal demarcation line is an indirect measure of riboflavin penetration in the stroma. Recent studies show that the demarcation line after I-CXL is rarely distinctly distinguished, with an average depth of 216 μ ± 49 mm.^[17]^

In line with these results, we can assume that I-CXL allows sufficient penetration of riboflavin in a very thin cornea.

Last but not least, a possible different behavior of the corneal epithelium when penetrated by riboflavin should be considered: the usual CXL is performed in early and moderate stages of disease when the epithelial basal layer is intact; in the advanced stages of the disease, the epithelial basal layer is altered which could alter its barrier function.^[27,28]^

To the best of our knowledge, this is the first study that evaluates this crosslinking method in keratoconic eyes with pachymetry of <400 μ.

It is difficult to compare the results of the studies due to various techniques, differences between the analyzed groups of patients, different methods and tools used for diagnosis and follow-up, methods of statistical analysis, etc.

For example, the comparison of 4 specular microscopes reveals different results in both the amount of endothelial cells required for examination and the ECD.^[29]^

Our results on UDVA and CDVA are similar to those of other studies that recorded the stabilization or improvement of these parameters at the end of the follow-up period.^[11,21,22,30]^

The K max value statistically decreased significantly from the sixth postoperative month to the last part of the follow-up period; our results are consistent with other studies.^[11,21,22,30]^

The K min values remained unchanged during the follow-up in our study. Cagil notes a decrease of this parameter values.^[21]^

Twelve months after surgery we observed a slight increase in CTTP (approximately 15 μ) that is statistically significant; no change in thickness was observed in other studies.^[11,21,22]^

The limits of our study are related to the retrospective nature, the small number of patients, and the short follow-up. To clarify the role of I-CXL in this situation, large prospective studies are needed with long-term follow-up of these patients.

## 6 Conclusions

Our study demonstrates the effectiveness of I-CXL in patients with progressive keratoconus and very thin corneas; our results are encouraging both to stabilize the disease and to improve some functional and refractive parameters with no side effects over a 12-month follow-up period. Applications of the procedure could be extended to patients in more advanced stages of keratoconus.
